# Time to split: biomarker trajectories in pediatric acute respiratory distress syndrome hint at underlying disease

**DOI:** 10.1172/JCI180662

**Published:** 2024-05-15

**Authors:** Ashley A. Zurawel, Bria M. Coates

**Affiliations:** 1 Ann & Robert H. Lurie Children’s Hospital of Chicago, Chicago, Illinois, USA.; 2 Northwestern University Feinberg School of Medicine, Chicago, Illinois, USA.

## Abstract

Pediatric acute respiratory distress syndrome (ARDS) is severe, noncardiac hypoxemic respiratory failure that carries a substantial risk of death. Given the complexity of this clinically defined syndrome and the repeated failure of therapeutic trials, there has been an effort to identify subphenotypes of ARDS that may share targetable mechanisms of disease. In this issue of the *JCI*, Yehya and colleagues measured 19 plasma biomarkers in 279 children over the first seven days of ARDS. Increases in select tissue injury makers and inflammatory cytokines in peripheral blood were associated with multiple organ dysfunction syndrome and death, but not persistent ARDS. These findings argue that splitting patients by clinical and molecular phenotype may be more informative than lumping them under the umbrella diagnosis of ARDS. However, future studies are needed to determine whether these plasma factors represent targetable pathways in lung injury or are a consequence of systemic organ dysfunction.

## A heterogeneous syndrome

Acute onset of severe hypoxemia that cannot be explained by cardiac dysfunction has been labelled acute respiratory distress syndrome (ARDS) and is associated with substantial morbidity and mortality in children and adults (1–3). Causes of ARDS are diverse, include direct and indirect mechanisms of lung injury, and vary across age groups (4–10). Therapeutic interventions for adult ARDS have been generally unsuccessful, prompting a movement to identify subgroups within this heterogeneous syndrome that may be more or less likely to respond to a given therapy. Given key differences in the epidemiology of pediatric and adult ARDS, and thus potential differences in underlying biology, it is important to characterize ARDS in the pediatric population (9, 11, 12).

**Figure 1:**
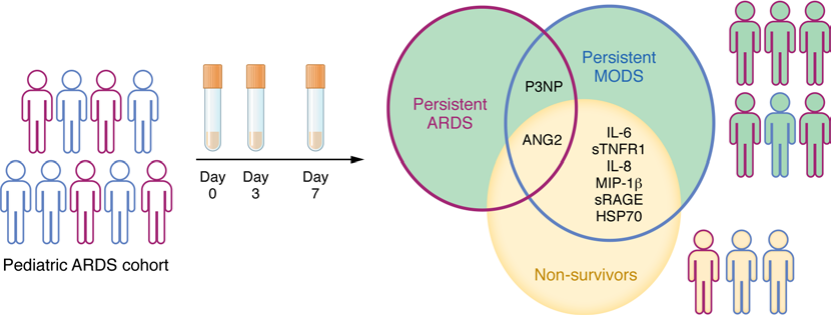
Pediatric patients with persistent ARDS possess biomarkers in common with persistent MODS and nonsurvivors. Yehya et al. (13) identified biomarkers in 279 patients with ARDS, in which 64 were nonsurvivors. Persistent ARDS was associated with P3NP and ANG2, whereas MODS was associated with multiple cytokines, tissue injury markers, and DAMPs.

In this issue of the *JCI*, Yehya et al. (13) described the longitudinal trajectory of several danger-associated molecular patterns (DAMPs), cytokines, and tissue injury markers in the blood of children with ARDS to identify subgroups that may share targetable mechanisms of disease. In this single-center prospective cohort study of 279 intubated and mechanically ventilated children with ARDS (as defined by the Berlin criteria), Yehya et al. (13) assessed biomarker profiles over a seven-day period following initial ARDS diagnosis. This approach builds on existing work utilizing plasma biomarkers to describe hypo- and hyperinflammatory subphenotypes of ARDS that may have differential risk of mortality and/or response to therapy (4, 5, 7, 8, 10, 11). Yehya and colleagues identified different biomarker trajectories in survivors compared with nonsurvivors, who showed early and persistent elevations in multiple DAMPs and tissue injury markers, and later increases in inflammatory cytokines and chemokines. Intriguingly, biomarkers characteristic of nonsurvivors more closely overlapped with biomarkers characteristic of multiple organ dysfunction syndrome (MODS), as opposed to persistent ARDS (13) (Figure 1). The pattern suggests this biomarker panel may be more reflective of systemic inflammation than lung injury. Given the repeated association between MODS and mortality in pediatric critical illness (14, 15), the biomarker overlap between nonsurvivors and persistent MODS is not surprising, especially since biomarkers were only measured in the peripheral blood, and not the lungs. However, the association does support the hypothesis that nonpulmonary organ dysfunction may be an important driver of mortality in patients with ARDS. The fact that many of the nonsurvivors appear to have had either resolved or nonsevere ARDS at the time of death supports this hypothesis.

## Mortality in patients with ARDS

Importantly, nonsurvivors in this cohort were more likely to be immunocompromised (*n* = 34/64 nonsurvivors [53%], *P* < 0.001) than survivors (*n* = 39/215 survivors [18%], *P* < 0.001). Immunocompromised participants had more nonpulmonary organ dysfunction at ARDS onset, were more likely to have nonpulmonary sepsis as an underlying etiology, and had higher levels of cytokines, chemokines, and tissue injury markers, mirroring the subgroup with MODS. While these biomarkers increased over time, it is unclear whether ARDS progression or evolving MODS caused these factors to increase. Interestingly, total biomarker levels, but not trajectories, were associated with death in the immunocompetent participants. In contrast, total levels of tissue injury markers and an increasing trajectory of those markers were associated with death in the immunocompromised participants (13). Future studies to determine whether this association reflects ongoing injury and/or a failure to resolve inflammation in immunocompromised patients are needed, as the pattern may represent an opportunity for therapeutic intervention.

Despite an improvement in supportive care, mortality rates in pediatric ARDS remain high and there are very few, if any, therapies available to target underlying mechanisms of disease. Recently, corticosteroids have been conditionally recommended by the Society of Critical Care Medicine and the American Thoracic Society to attenuate the inflammatory response in adult ARDS (16), but conflicting adult data and a lack of evidence in pediatric ARDS make it unclear how or whether corticosteroids should be used in children (17). In this cohort, approximately half of participants received corticosteroids and rates of use were not significantly different between survivors and nonsurvivors (*n* = 103/215 survivors [48%] versus *n* = 39/64 nonsurvivors [61%], *P* = 0.159). However, participants who received corticosteroids had more severe lung disease (oxygen index 12.9 versus 10.1, *P* < 0.002), suggesting providers may be more likely to reach for corticosteroids for the sickest patients. Several biomarkers were decreased in participants who received corticosteroids compared with those who did not, including interleukin 6, soluble tumor necrosis factor receptor 1, matrix metalloprotease 8, macrophage inhibitor protein 1β, and procollagen type III N-terminal peptide, but it is impossible to know whether these changes were a direct effect of corticosteroids or secondary to other confounders. In addition, while these proteins were associated with mortality in the entire cohort, they were not associated with mortality in the group that did not receive corticosteroids.

## Conclusions

The stated goal of Yehya et al. (13) was to describe the longitudinal molecular signature of pediatric ARDS with the hope that it would provide insight into mechanisms driving disease. The collection of serial samples in this large cohort of children is impressive and is an important first step in characterizing inflammation in children with ARDS. However, it remains to be seen whether a peripheral blood biomarker profile can provide insights into the mechanisms driving the heterogeneous syndrome that is ARDS in the lungs. In future studies, it will be important to tease apart markers of nonpulmonary organ dysfunction from markers of lung injury, and to separate markers of established injury from drivers of ongoing tissue damage that can be interrupted.

Finally, Yehya et al.’s finding that mortality in their cohort was more closely aligned with the extent of nonpulmonary organ dysfunction than persistent ARDS suggests that continuing to use the diagnosis of ARDS for inclusion in clinical trials may be misguided (13). The extensive efforts to subphenotype, or split, syndromes defined by clinical characteristics into treatable diseases begs the question: Is it time to abandon the artificial groupings of patients by symptoms?

## Version 1 05/15/2024

Electronic publication

## References

[1] Ashbaugh DG (1967). Acute respiratory distress in adults. Lancet.

[2] Force ADT (2012). Acute respiratory distress syndrome: the Berlin definition. JAMA.

[4] Calfee CS (2014). Subphenotypes in acute respiratory distress syndrome: latent class analysis of data from two randomised controlled trials. Lancet Respir Med.

[5] Famous KR (2017). Acute respiratory distress syndrome subphenotypes respond differently to randomized fluid management strategy. Am J Respir Crit Care Med.

[6] Sinha P (2020). Machine learning classifier models can identify acute respiratory distress syndrome phenotypes using readily available clinical data. Am J Respir Crit Care Med.

[7] Sinha P (2022). Latent class analysis-derived subphenotypes are generalisable to observational cohorts of acute respiratory distress syndrome: a prospective study. Thorax.

[8] Sinha P (2020). Development and validation of parsimonious algorithms to classify acute respiratory distress syndrome phenotypes: a secondary analysis of randomised controlled trials. Lancet Respir Med.

[9] Dahmer MK (2022). Identification of phenotypes in paediatric patients with acute respiratory distress syndrome: a latent class analysis. Lancet Respir Med.

[10] Flori HR (2023). Subphenotypes assigned to pediatric acute respiratory failure patients show differing outcomes. Am J Respir Crit Care Med.

[11] Ardila SM (2023). A targeted analysis of serial cytokine measures and nonpulmonary organ system failure in children with acute respiratory failure: individual measures and trajectories over time. Pediatr Crit Care Med.

[12] Khemani RG (2019). Paediatric acute respiratory distress syndrome incidence and epidemiology (PARDIE): an international, observational study. Lancet Respir Med.

[13] Yehya NB (2024). Inflammatory and tissue injury marker dynamics in pediatric acute respiratory distress syndrome. J Clin Invest.

[14] Watson RS (2017). Epidemiology and outcomes of pediatric multiple organ dysfunction syndrome. Pediatr Crit Care Med.

[15] Badke CM (2022). Multiple organ dysfunction interactions in critically ill children. Front Pediatr.

[16] Qadir N (2024). An update on management of adult patients with acute respiratory distress syndrome: an official American Thoracic Society clinical practice guideline. Am J Respir Crit Care Med.

[17] Yehya N (2015). Corticosteroid exposure in pediatric acute respiratory distress syndrome. Intensive Care Med.

